# Supplementary material for: Epidemiological characteristics of multidrug-resistant Acinetobacter baumannii ST369 in Anhui, China
Source: mSystems. 2023 Sep 1;8(5):e00731-23. doi: 10.1128/msystems.00731-23 (PMC10654100; doi:10.1128/msystems.00731-23)
Supplement: Supplemental figures — Figures S1 to S4. [file msystems.00731-23-s0001.docx]

**Epidemiological characteristics of multidrug-resistant *Acinetobacter baumannii* ST369 in Anhui, China**

Yi Huang^a,b^, Md Roushan Ali^b^, Wei Li^a,b^, Wanying Wang^c^, Yuanyuan Dai^a^, Huaiwei Lu^a^, Zhien He ^*,a,b^, Yujie Li ^*,a,b^ and Baolin Sun ^*,a,b^

*Corresponding Authors

Baolin Sun*: [sunb@ustc.edu.cn](mailto:sunb@ustc.edu.cn).

Zhien He*: [zhienhe@mail.ustc.edu.cn](mailto:zhienhe@mail.ustc.edu.cn).

Yujie Li*: [lyj2020@ustc.edu.cn](mailto:lyj2020@ustc.edu.cn)

Yi Huang: First author, [huang420@mail.ustc.edu.cn](mailto:huang420@mail.ustc.edu.cn).

**^a^**Department of Oncology, The First Affiliated Hospital of Division of Life Sciences and Medicine, University of Science and Technology of China, Hefei, Anhui Province 230026, China.

**^b^**Division of Life Sciences and Medicine, University of Science and Technology of China, Hefei, Anhui Province 230026, China.

**^c^**Intensive Care Unit, Biomedical Research Center, Shenzhen Institute of Translational Medicine, Health Science Center, The First Affiliated Hospital of Shenzhen University, Shenzhen Second People’s Hospital, Shenzhen 518035, China.

**Supplementary Figures**


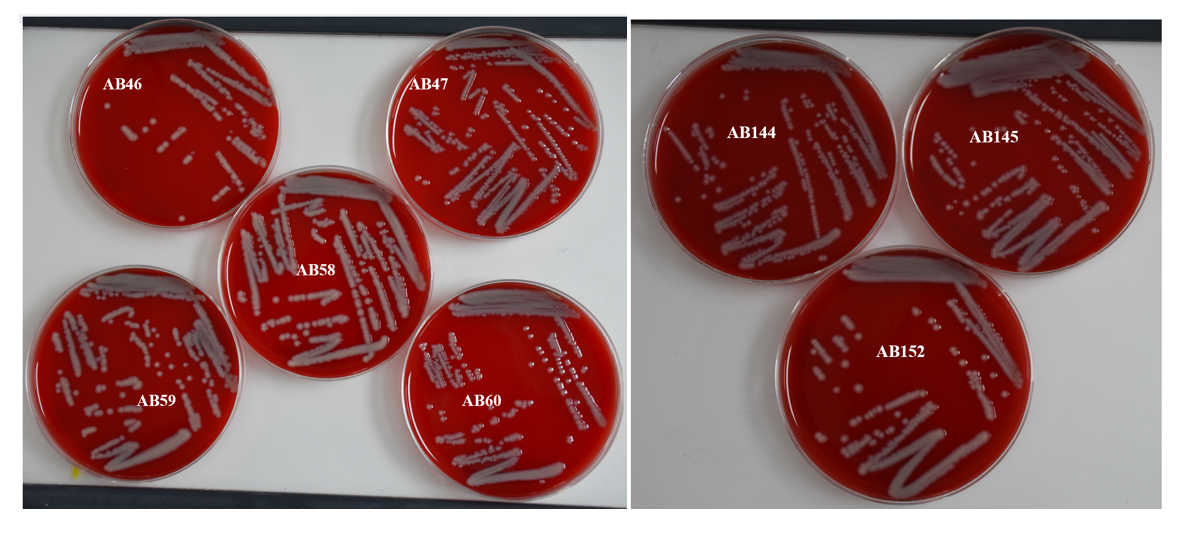


Figure S1: Colony morphology of 8 isolates. The isolates were inoculated on fresh blood plates and cultured overnight at 37°C. The monoclonal morphology of the isolates was observed the following day.


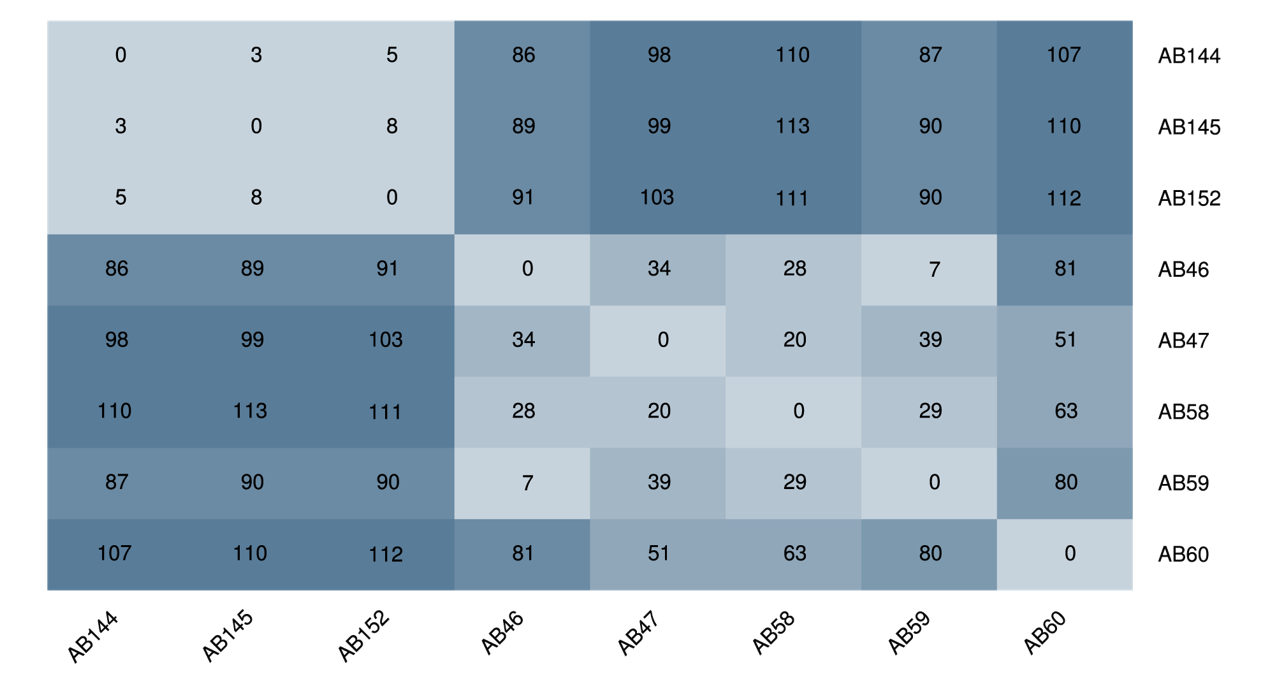


Figure S2 SNP analysis of 8 isolates. SNP analysis revealed that AB46, AB47, AB58, AB59, and AB60 SNPs exhibited minor variations, whereas AB144, AB145, and AB152 showed similar small differences.


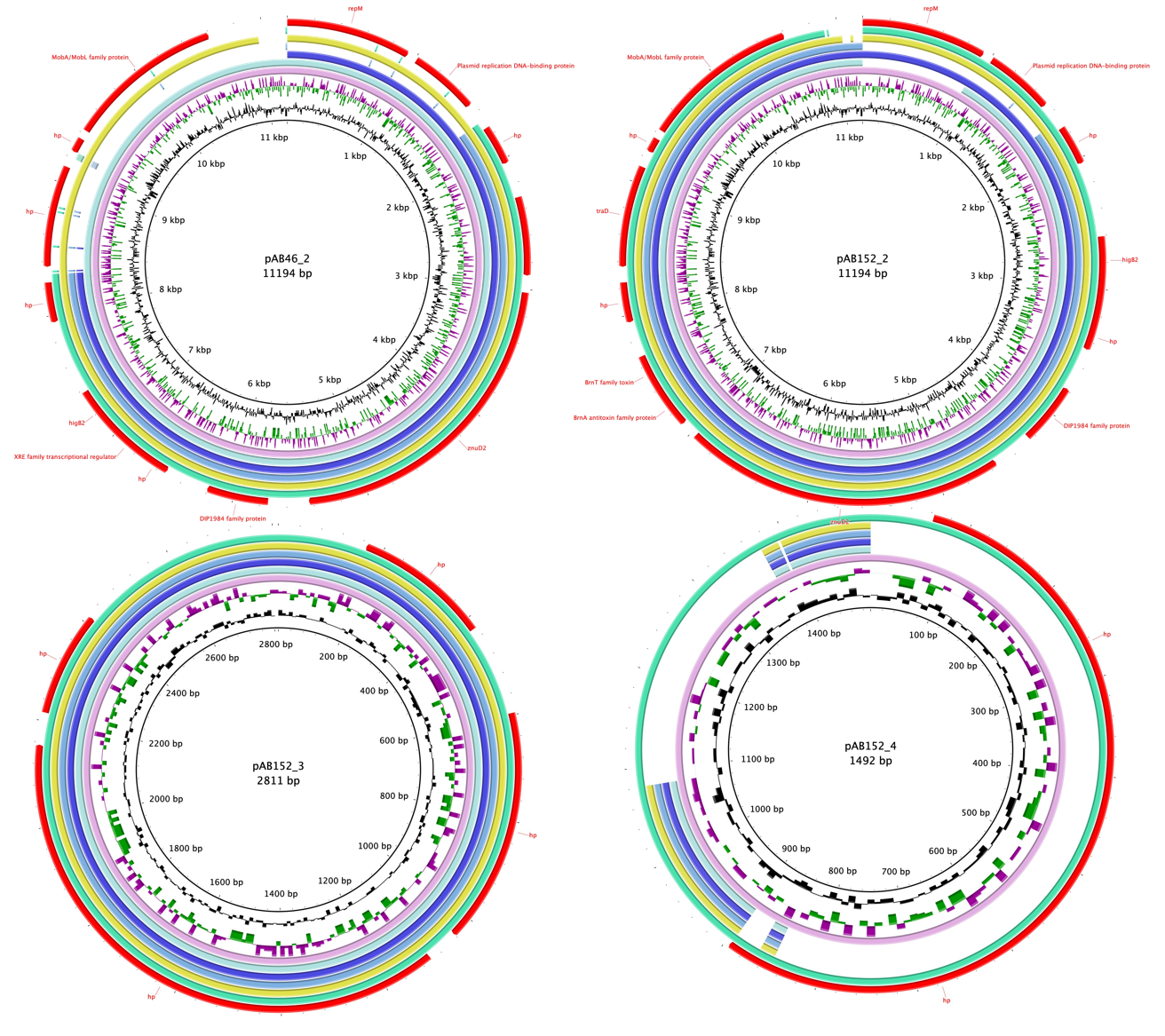


Figure S3 Plasmids of AB46 and AB152. We performed genome assembly on the three-generation sequencing results of AB46 and AB152, and identified some small plasmids. The red label represents the gene we identified in these plasmids.


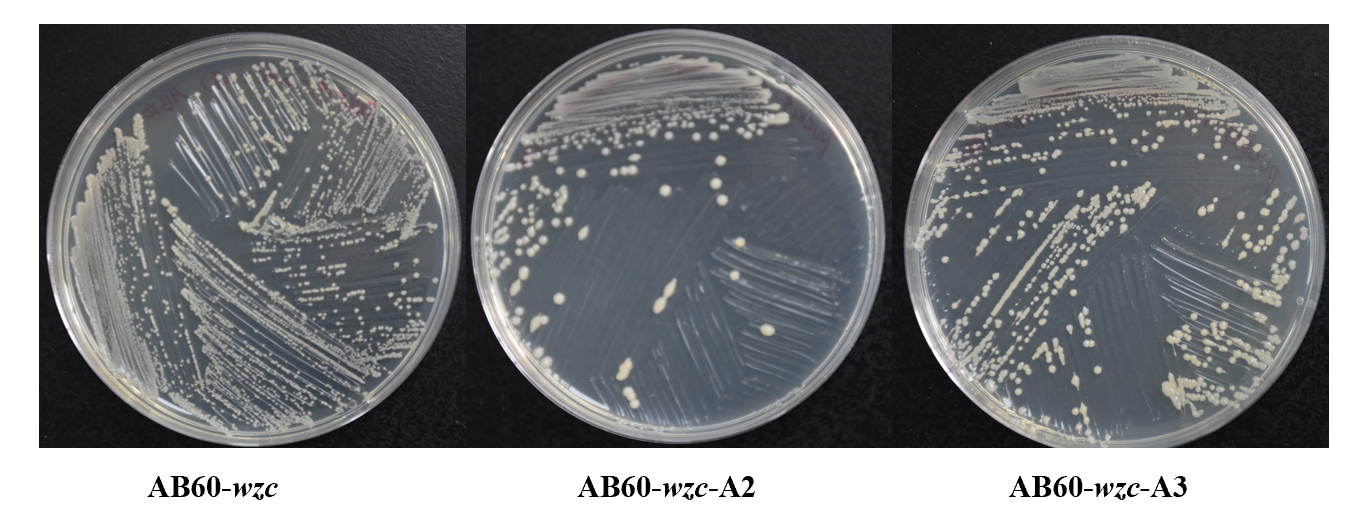


Figure S4 Colony morphology of AB60-*wzc*, AB60-*wzc*-A2, AB60-*wzc*-A3. Isolates were inoculated on fresh LB plates and cultured overnight at 37°C. After inserting pUCk19-*wzc* (site mutation on 540 and 667), the clones become more sticky.
